# Supplementary material for: The chromatin-associated lncREST ensures effective replication stress response by promoting the assembly of fork signaling factors
Source: Nat Commun. 2024 Feb 1;15:978. doi: 10.1038/s41467-024-45183-5 (PMC10834948; doi:10.1038/s41467-024-45183-5)
Supplement: Supplementary file 1 — Supplementary Information [file 41467_2024_45183_MOESM1_ESM.pdf]

The chromatin-associated *lncREST* ensures effective replication stress response by promoting the assembly of fork signaling factors

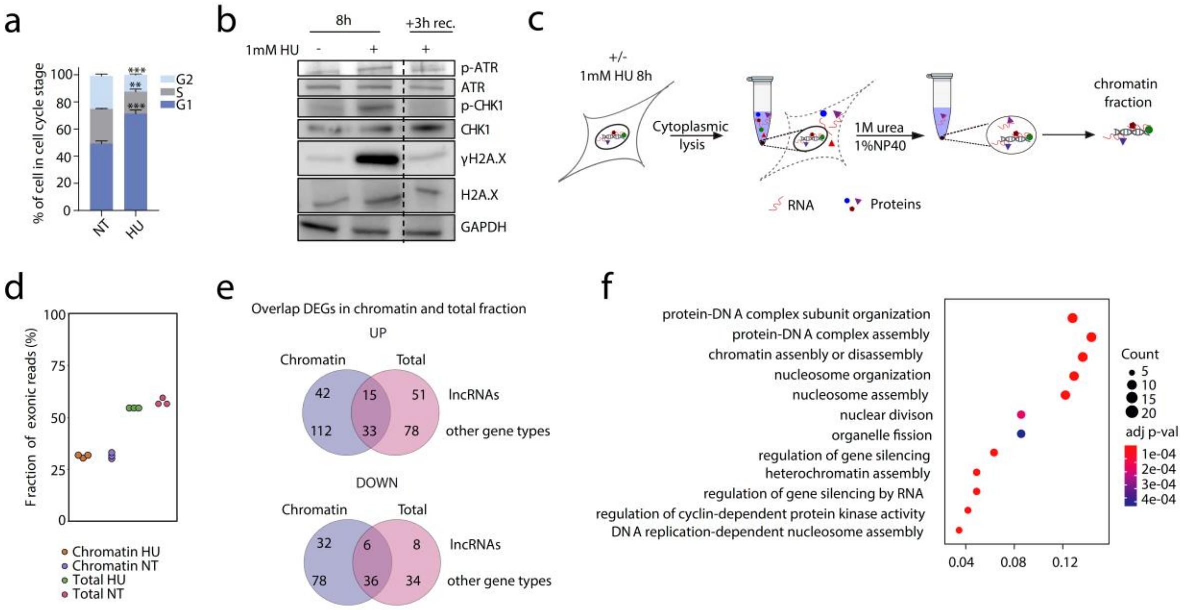

Supplementary Figure 1. Replication stress induces the expression of RNAs involved in regulation of genome stability

(a) Analysis of cell-cycle phase distribution by flow cytometry of propidium iodide-stained HCT116 cells treated with 1mM HU for 8hrs and relative untreated control. G1 **p=0.000321, S **p=0.0013, G2 ***p=0.00022 (mean ± SD, n = 3). (b) Immunoblot analysis HCT116 cells treated with HU 1mM for 8hrs followed by 3h recovery shows the reversible effect of HU on replication stress markers p-ATR, p-CHK1, γH2A.X and total counterparts. Source data are provided as Source Data file. (c) Schematic of the experimental workflow of cell fractionation. (d) Fraction of reads that match an annotated GENCODE exon for each one of the RNA-seq conditions. (e) Venn diagram showing the overlap of differentially expressed genes in the chromatin and total RNA fraction. Numbers on the top indicate lncRNAs, numbers on the bottom indicate other gene types. (f) Panther GO-term enrichment analysis of the differentially expressed protein-coding genes in the chromatin fraction.

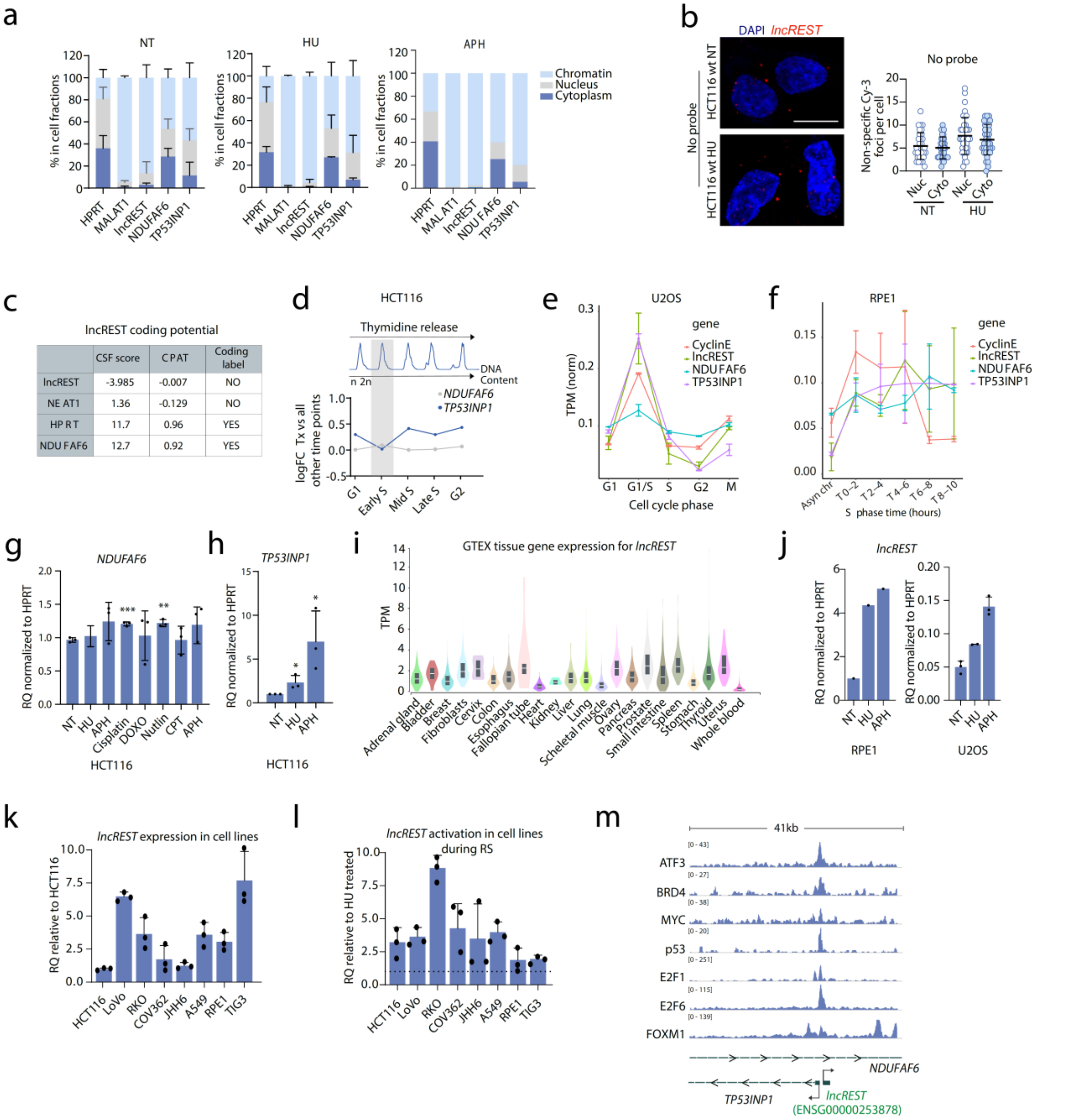

Supplementary Figure 2. Identification and characterization of the lncRNA *lncREST*.

(a) Relative subcellular localization of *lncREST* and its neighbor genes *NDUFAF6* and *TP53INP1* with respect to the control RNAs *HPRT* and *MALAT1* following HU induction (1mM o.n.) or 400mM Aphidicolin (APH) 24 hrs, determined by nucleus/cytoplasm fractionation of HCT116 followed by qRT-PCR. (b) Representative images of no probe control FISH in HCT116 cells treated with PBS or

HU o.n. and relative quantification of unspecific nuclear and cytoplasmic Cy-3 signal. Scale bar: 10µm. 30 cells were analyzed per replicate, n=2 (c) Table indicating the noncoding capacity of lncREST using two different tools, with respect to control coding and non-coding RNAs. (d) From top to bottom: Flow cytometry profiles of HCT116 synchronized cells, representing the DNA content at different cell cycle stages from release; relative expression of *NDUFAF6* and *TP53INP1* measured by RNA-seq. log2FC of each time point vs all other time points are represented. DESeq two-sided, with Benjamini-Hochberg FDR correction. (e and f) Relative expression of lncREST, *Cyclin E*, *NDUFAF6* and *TP53INP1* at different cell cycle stages measured by RNA- seq in U2OS (e) and RPE1 (f). (g) qRT-PCR analysis of *NDUFAF6* expression in HCT116 cells treated with 2mM of hydroxyurea (HU) o.n., 15µM of cisplatin (cis) o.n., 5 µM of doxorubicin (Dox) for 24 hrs, 20 µM of nutlin (Nut) o.n., 10 µM of camptotecin (CPT) for 8 hrs, aphidicolin (APH) 40 µM for 24 hrs relative to normal condition (NT). N= 3 biological replicates. **p < 0.01, ***p < 0.001, ****P<0.0001 (mean ± SD, two-tailed unpaired t-test). (h) qRT-PCR analysis of *TP53INP1* expression in HCT116 cells treated with 2mM of hydroxyurea (HU) o.n and 400µM Aphidicolin (APH) 24 hrs. (i) Bulk tissue lncREST expression retrieved from GTEX database (https://gtexportal.org). Expression values are shown in TPM (Transcripts Per Million). (j) qRT-PCR analysis of *lncREST* induction in RPE1 and U2OS cells treated with 2mM of hydroxyurea (HU) o.n and 400µM Aphidicolin (APH) 24hrs. (k and l) qRT-PCR of *lncREST* in different human cancer and normal cell lines, with respect to HCT116 cells in non-treated conditions (k), and in HU treated (2 mM o.n.) with respect to non-treated condition for each cell line (l). Dotted line represents the RQ value of the reference non-treated sample (set as 1). (m) Integrative genomics viewer (IGV) browser snapshot of ChIP-seq tracks of different stress-related transcription factors retrieved from Enrichr software showing their association with *lncREST* promoter. For a, b, d, g, h, j, k and l Source data are provided as a Source Data file.

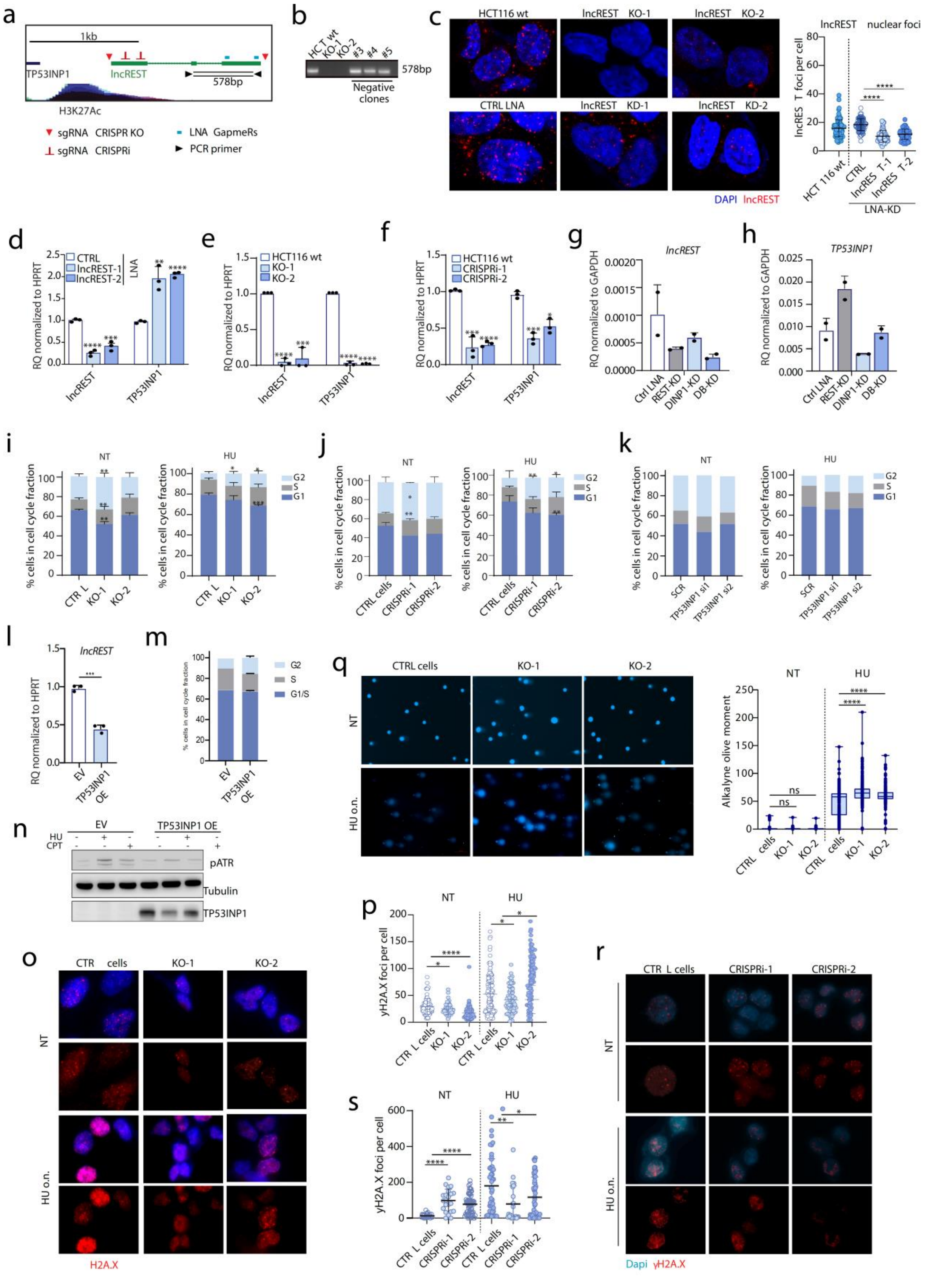

a
1kb
TP53INP1
lncREST
578bp
H3K27Ac
sgRNA CRISPR KO
sgRNA CRISPRi
LNA GapmeRs
PCR primer
b
HCT wt
KO-1
KO-2
#3
#4
#5
578bp
Negative clones
c
HCT116 wt
lncREST KO-1
lncREST KO-2
CTRL LNA
lncREST KD-1
lncREST KD-2
DAPI lncREST
lncREST nuclear foci
lncREST foci per cell
HCT 116 wt
CTRL
lncREST-1
lncREST-2
LNA-KD
d
CTRL
lncREST-1
lncREST-2
LNA
RQ normalized to HPRT
lncREST
TP53INP1
e
HCT116 wt
KO-1
KO-2
RQ normalized to HPRT
lncREST
TP53INP1
f
HCT116 wt
CRISPRi-1
CRISPRi-2
RQ normalized to HPRT
lncREST
TP53INP1
g
lncREST
RQ normalized to GAPDH
Ctrl LNA
REST-KD
DINP1-KD
DB-KD
h
TP53INP1
RQ normalized to GAPDH
Ctrl LNA
REST-KD
DINP1-KD
DB-KD
i
NT
HU
% cells in cell cycle fraction
CTRL
KO-1
KO-2
G2
S
G1
j
NT
HU
% cells in cell cycle fraction
CTRL cells
CRISPRi-1
CRISPRi-2
G2
S
G1
k
NT
HU
% cells in cell cycle fraction
SCR
TP53INP1 si1
TP53INP1 si2
G2
S
G1
l
lncREST
RQ normalized to HPRT
EV
TP53INP1 OE
m
% cells in cell cycle fraction
G2
S
G1/S
EV
TP53INP1 OE
n
EV
TP53INP1 OE
HU
CPT
pATR
Tubulin
TP53INP1
q
CTRL cells
KO-1
KO-2
NT
HU o.n.
NT
HU
Alkalyne olive moment
ns
CTRL cells
KO-1
KO-2
CTRL cells
KO-1
KO-2
p
NT
HU
γH2A.X foci per cell
CTRL cells
KO-1
KO-2
CTRL cells
KO-1
KO-2
o
CTRL cells
KO-1
KO-2
NT
HU o.n.
H2A.X
r
CTRL cells
CRISPRi-1
CRISPRi-2
NT
HU o.n.
Dapi γH2A.X
s
NT
HU
γH2A.X foci per cell
CTRL cells
CRISPRi-1
CRISPRi-2
CTRL cells
CRISPRi-1
CRISPRi-2

Supplementary Figure 3. *lncREST* KD phenotypes are independent of the neighbor protein coding gene *TP53INP1*.

(a) Diagram of the strategies applied to deplete *lncREST*, indicating the target sequences for the LNAs and the positions of the dCas9/KRAB and CRISPR KO sgRNAs and primers to detect the KO. (b) Genotypes of HTC116 wt and KO clones were detected by PCR of a 578 bp region of *lncREST*. (c) Representative images of *lncREST* FISH in HCT116 wt, *lncREST* KO and *lncREST* depleted cells with LNA GapmeRs and dot plot of *lncREST* foci quantification, mean ± STD. Three experiments were performed with similar results, at n=60 cells per condition were analyzed. ****p < 0.0001 (two-tailed unpaired t-test). (d-f) qRT-PCR showing the efficiency of *lncREST* KD or KO and the expression of *TP53INP1*, in HCT116 cells transfected with LNAs, in CRISPR KO and DCas9/KRAB cells. **p<0.01, ***p<0.001, ****p<0.0001 (two-tailed unpaired t-test. n = 3 per group). (g and h) Expression levels of *lncREST* and *TP53INP1* in untreated cells where *lncREST* in depleted with LNA (lncREST-KD), *TP53INP1* is depleted with siRNA (TP53INP1-KD) or both (Double-KD). Histograms represent data obtained from n=2 experiments. (i-k) Analysis of cell-cycle phase distribution by flow cytometry of propidium iodide- stained HCT116 CRISPR KO (i), CRISPRi (j) and *TP53INP1* KD cells (k) treated with 1mM HU for 8h and relative untreated control. *p<0.05, **p<0.01, ***p<0.001 (two tailed unpaired t-test, mean ± SD, n = 3 per group). (l) qRT-PCR of *lncREST* expression in *TP53INP1* overexpressing cells **p<0.01, ***p<0.001, ****p<0.0001 (two-tailed unpaired t-test. n = 3 per group). (m) Analysis of cell-cycle phase distribution by flow cytometry of propidium iodide-stained HCT116 overexpressing *TP53INP1*. The graph shows data obtained from n=2 experiments. (n) WB indicating the levels of p-ATR, and TP53INP1 in control HCT116 cells (EV) or overexpressing *TP53INP1*. Cells are not treated or treated with CTP or HU as indicated. (o, p) Representative images of gH2A.X foci in two independent clones of *lncREST* CRISPR KO cells in NT and HU conditions (o) and relative quantification shown by dot plot and mean of gH2A.X foci per nucleus (p). 100 were analyzed per sample, n=2 (Mann Whitney Wilcoxon U-test). *p<0.05, **p<0.01, ****p<0.0001. Scale bar, 10 µm. (q) Alkaline comet assay showing the increase ss-and dsDNA breaks in *lncREST* CRISPR KO cells, HU treated as indicated. Upper panel, representative images. Lower panel, quantification of alkaline olive moment. At least 150 tails were analyzed in each group. Whiskers are set to min/max value, median is shown.

****p<0.0001, two-tailed unpaired t-test. n = 3. Scale bar, 100µm. (r, s) Representative images of γH2A.X foci in two independent clones of lncREST dCas9/KRAB cells in NT and HU conditions (r) and relative quantification by dot plot and mean of gH2A.X foci per nucleus (s). One hundred cells were analyzed per sample, n=2. (Mann Whitney Wilcoxon U-test). *p<0.05, **p<0.01, ****p<0.0001. Scale bar, 10 µm. For a-s Source data are provided as a Source Data file.

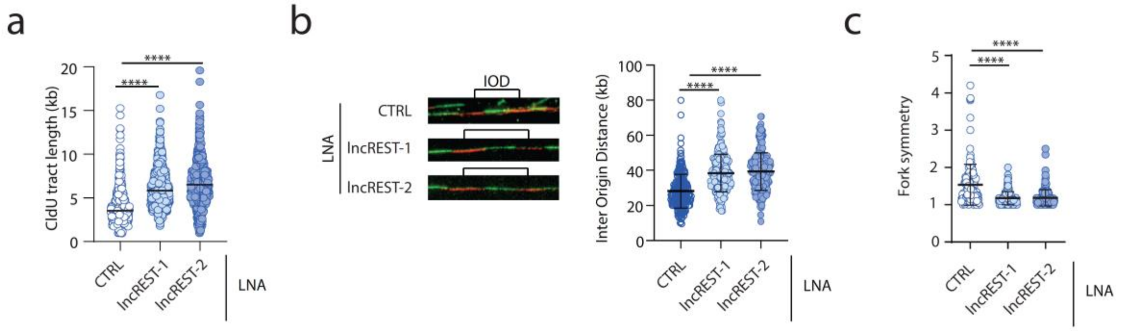

Supplementary Figure 4. ***lncREST*****-associated DNA fiber phenotypes**

(a) Dot blot and median of IdU tracts in HCT116 treated with control or *lncREST* Knockdown. ****p<0.0001, by Mann-Whitney test. (b) Inter origin distance calculated as the distance between two CldU (green) tracts separated by an IdU (red) tract, in HCT116 treated with control or lncREST Knockdown. ****p<0.0001, by Mann-Whitney test. (c) Fork symmetry analysis in Control and lncREST KD cells, calculated as the ratio between two CldU tracts separated by one IdU tract. *p<0.05, **p<0.01 by Mann- Whitney test. 150 signals were analyzed from each condition (n=3).

Source data are provided as a Source Data file.

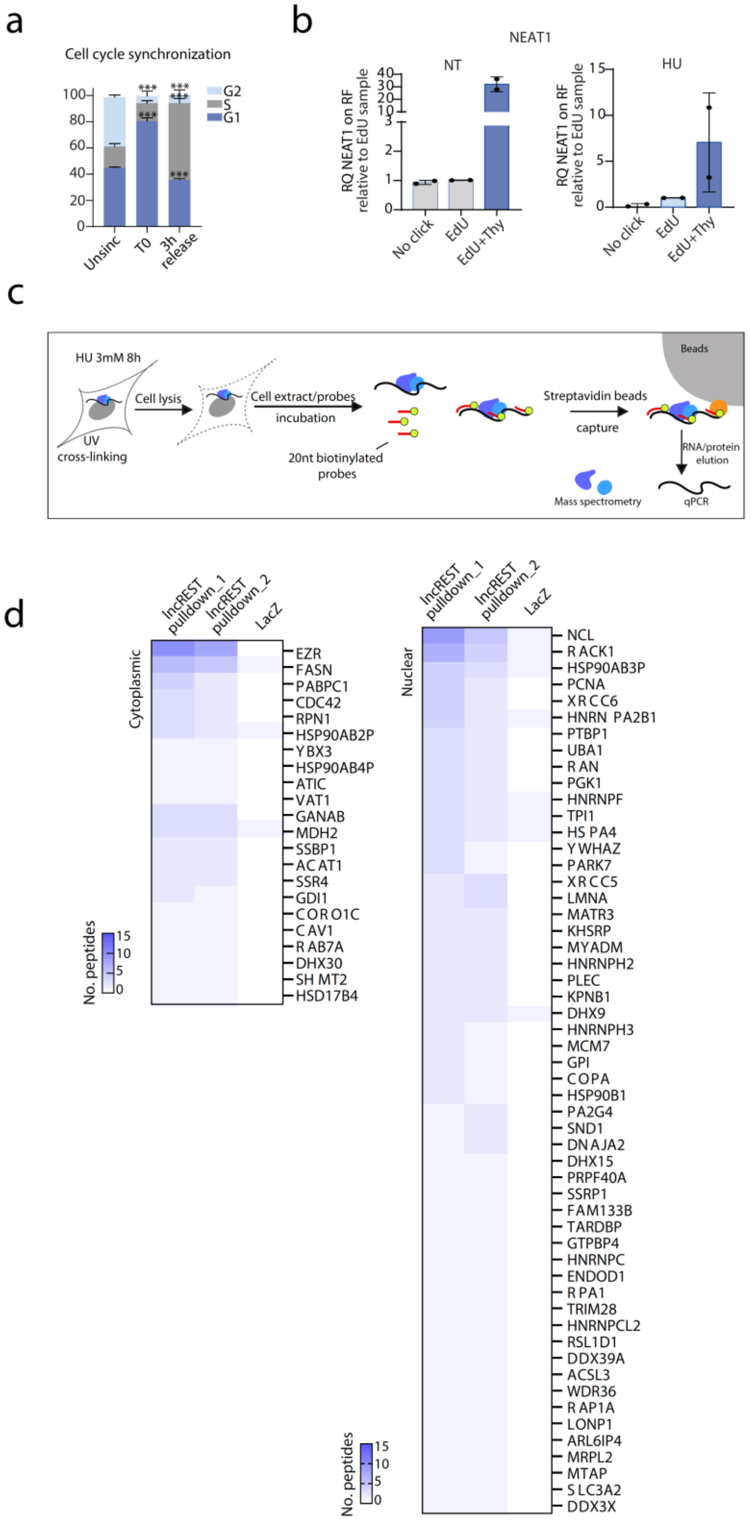
a
Cell cycle synchronization
G2
S
G1
Unsinc
T0
3h release
b
NEAT1
NT
HU
RQ NEAT1 on RF relative to EdU sample
No click
EdU
EdU+Thy
c
HU 3mM 8h
UV cross-linking
Cell lysis
Cell extract/probes incubation
20nt biotinylated probes
Streptavidin beads capture
Beads
RNA/protein elution
Mass spectrometry
qPCR
d
lncREST pulldown_1
lncREST pulldown_2
LacZ
Cytoplasmic
No. peptides
EZR
FASN
PABPC1
CDC42
RPN1
HSP90AB2P
YBX3
HSP90AB4P
ATIC
VAT1
GANAB
MDH2
SSBP1
ACAT1
SSR4
GDI1
CORO1C
CAV1
RAB7A
DHX30
SHMT2
HSD17B4
Nuclear
NCL
RACK1
HSP90AB3P
PCNA
XRCC6
HNRNPA2B1
PTBP1
UBA1
RAN
PGK1
HNRNPF
TPI1
HSPA4
YWHAZ
PARK7
XRCC5
LMNA
MATR3
KHSRP
MYADM
HNRNPH2
PLEC
KPNB1
DHX9
HNRNPH3
MCM7
GPI
COPA
HSP90B1
PA2G4
SND1
DNAJA2
DHX15
PRPF40A
SSRP1
FAM133B
TARDBP
GTPBP4
HNRNPC
ENDOD1
RPA1
TRIM28
HNRNPCL2
RSL1D1
DDX39A
ACSL3
WDR36
RAP1A
LONP1
ARL6IP4
MRPL2
MTAP
SLC3A2
DDX3X

Supplementary Figure 5. *lncREST* interacts with a number of nuclear proteins during replication stress

(a) Cell cycle analysis of HCT116 cells synchronized and released in S-phase before performing the iPOND experiments. ***p<0.001 (mean ± SD, two-tailed unpaired t-test. n = 3 per group). Source data are provided as a Source Data file. (b) qRT-PCR showing the amount of *NEAT1* relative to the EdU sample (nascent chromatin) in NT (left) and HU treated (right) samples. Each sample was normalized to its input (mean ± SD, n = 2). Source data are provided as a Source Data file. (c) Schematic workflow of the in vivo RNA pulldown experiment. (d) Heatmap showing the lists of nuclear and cytoplasmic protein interactors of *lncREST*, indicating the number of peptides identified in the two independent replicates relative to LacZ probe.

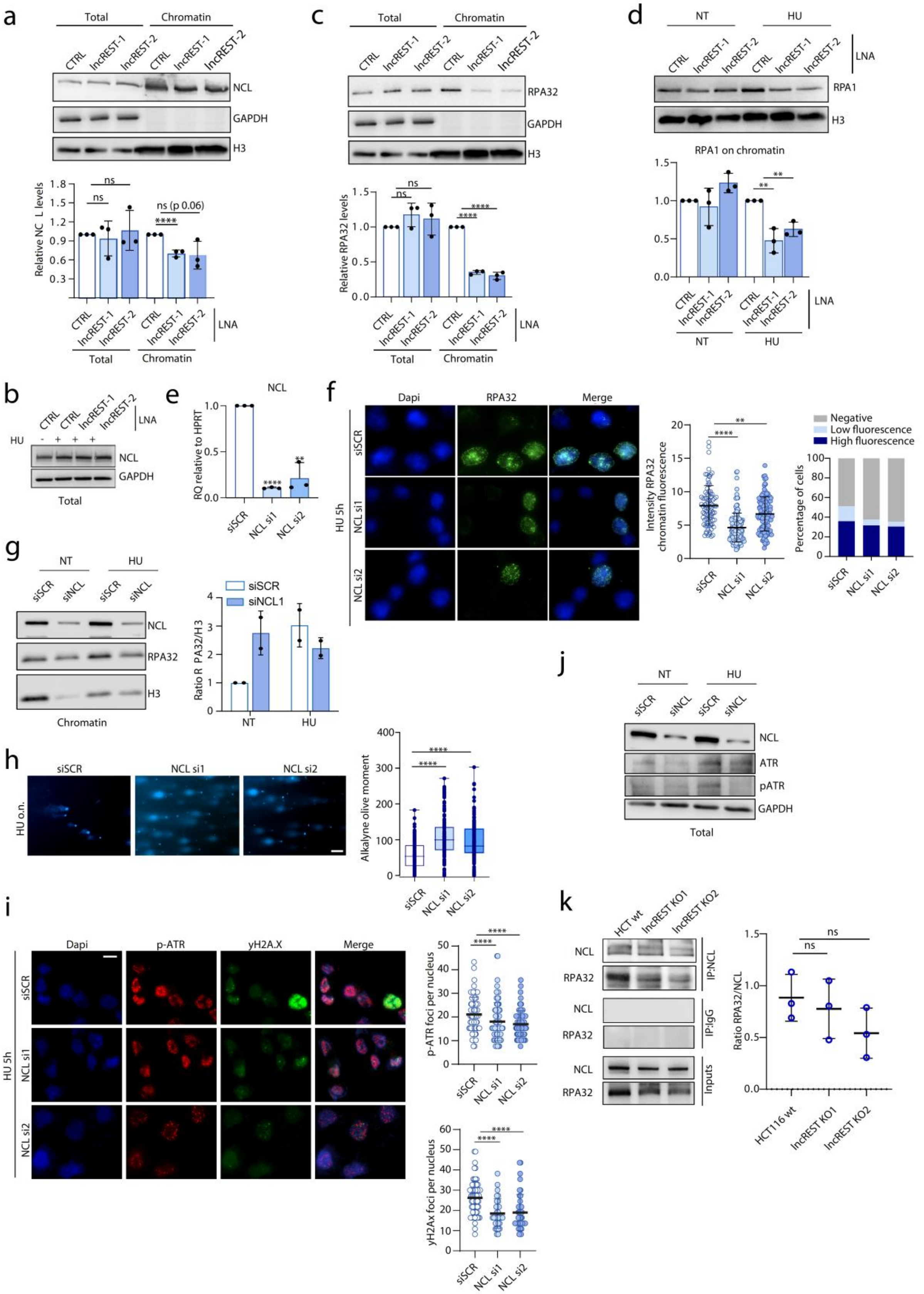
a
Total
Chromatin
CTRL
lncREST-1
lncREST-2
NCL
GAPDH
H3
Relative NC L levels
ns
ns (p 0.06)
****
LNA
b
HU
NCL
GAPDH
Total
c
RPA32
Relative RPA32 levels
d
NT
HU
RPA1
RPA1 on chromatin
e
NCL
RQ relative to HPRT
siSCR
NCL si1
NCL si2
f
Dapi
RPA32
Merge
HU 5h
Intensity RPA32 chromatin fluorescence
Negative
Low fluorescence
High fluorescence
Percentage of cells
g
siNCL
siNCL1
Chromatin
Ratio R PA32/H3
h
HU o.n.
Alkalyne olive moment
i
p-ATR
yH2A.X
p-ATR foci per nucleus
yH2Ax foci per nucleus
j
ATR
pATR
k
HCT wt
lncREST KO1
lncREST KO2
IP:NCL
IP:IgG
Inputs
Ratio RPA32/NCL
HCT116 wt

Supplementary Figure 6. *NCL* knockdown recapitulates *lncREST* depletion phenotypes during replication stress

(a) Western blot (upper) and quantification (lower) of NCL in total and chromatin fractions of CTRL and *lncREST* KD HCT116 cells in untreated and HU treated condition. **p<0.01 (two-tailed unpaired t-test. n = 3 per group). (b) Western blot of NCL in NT and HU treated conditions transfected with control (CTRL) or lncREST LNA. (c, d) Western blot (upper) and quantification (lower) of RPA32 (C) and RPA1 (D) in total and chromatin fractions of CTRL and *lncREST* KD HCT116 cells in untreated and HU treated condition. **p<0.01 (two-tailed unpaired t-test. n = 3 per group). (e) qRT-PCR detection of *NCL* following transfection of HCT116 cells with two independent siRNAs. **p<0.01, ***p<0.001 (mean ± SD, two-tailed unpaired t-test. n= 3). (f) Immunofluorescence of chromatin associated RPA32 in siSCR and *NCL* KD HCT116 cells following HU treatment, after removal of soluble cellular fraction before fixing the cells (upper), dot blot of RPA32 fluorescence (lower left), and percentage of cells positive to RPA32 (lower right). (Two-tailed unpaired t-test. n=3) **p<0.01, ****p<0.0001. (g) Western blot (left) and quantification (right) of RPA32 in chromatin fraction of CTRL and *NCL* KD HCT116 cells in untreated and HU treated condition (n=2). (h) Alkaline comet assay showing the increase ss-and dsDNA breaks in *NCL* KD cells, HU treated as indicated. Upper panel, representative images. Lower panel, quantification of alkaline olive moment. 150 tails were analyzed in each group, n=3. Whiskers are set to min/max value, median is shown. ****p<0.0001, two-tailed unpaired t-test. n = 3. Scale bar, 100µm. (i) Immunofluorescence of p-ATR and yH2AX in siSCR and *NCL* KD HU treated HCT116 (upper) and relative quantification of p-ATR and gH2A.X foci (lower). ****p<0.0001. C and D, mean ± SD, two-tailed unpaired t-test. n =3). (j) Western blot of the indicated proteins in the conditions shown in I. Representative images from two independent experiments. (k) Western blot (left) and quantification (right) of CTRL LNA or *lncREST* KO cells- NCL co- immunoprecipitated with RPA32 or a control IgG in untreated or HU treated cells, (mean ± SD, two-tailed unpaired t-test). n=3. For a, c-i and k Source data are provided as a Source Data file.

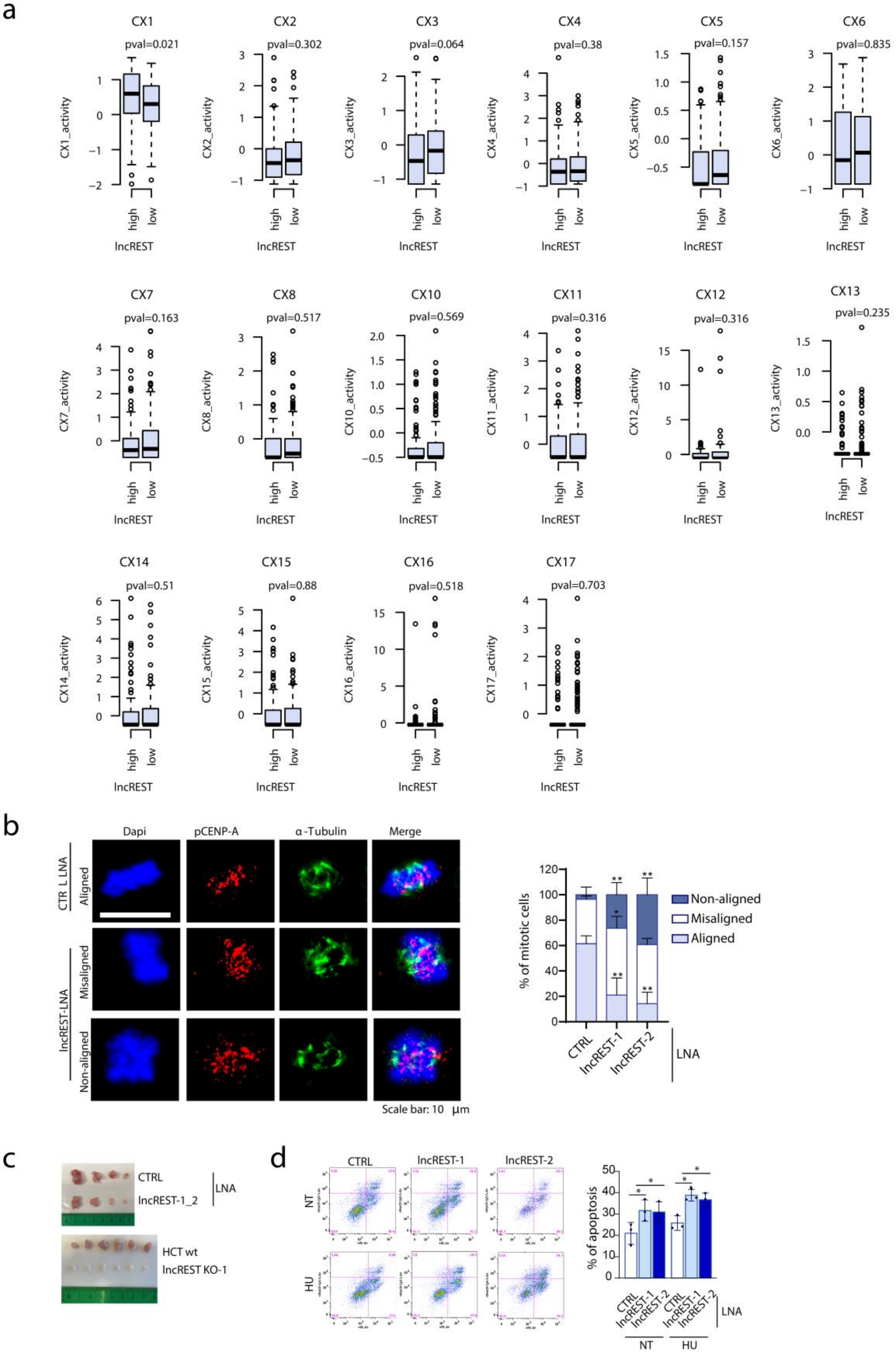
a
CX1
pval=0.021
CX2
pval=0.302
CX3
pval=0.064
CX4
pval=0.38
CX5
pval=0.157
CX6
pval=0.835
CX7
pval=0.163
CX8
pval=0.517
CX10
pval=0.569
CX11
pval=0.316
CX12
pval=0.316
CX13
pval=0.235
CX14
pval=0.51
CX15
pval=0.88
CX16
pval=0.518
CX17
pval=0.703
CX1_activity
CX2_activity
CX3_activity
CX4_activity
CX5_activity
CX6_activity
CX7_activity
CX8_activity
CX10_activity
CX11_activity
CX12_activity
CX13_activity
CX14_activity
CX15_activity
CX16_activity
CX17_activity
high
low
lncREST
b
Dapi
pCENP-A
α -Tubulin
Merge
CTR L LNA
Aligned
lncREST-LNA
Misaligned
Non-aligned
Scale bar: 10 µm
% of mitotic cells
Non-aligned
Misaligned
Aligned
CTRL
lncREST-1
lncREST-2
LNA
c
CTRL
lncREST-1_2
LNA
HCT wt
lncREST KO-1
d
CTRL
lncREST-1
lncREST-2
NT
HU
% of apoptosis
CTRL
lncREST-1
lncREST-2
LNA
NT
HU

Supplementary Figure 7. *lncREST* regulates cancer progression and apoptosis

(a) Score of the replication stress CX9 signature39 in TCGA colorectal tumors with high and low lncREST expression. TCGA patient samples were divided in two groups depending on their *lncREST* expression (40% upper percentile as high, 60% lower percentile as low). For each one of them, the activity of the 17 copy number signatures was calculated and plotted, as described in Drews et al., 2022. (b) Mitotic cells stained with α-tubulin (green) and p-CENP-A (red) antibodies in HCT116 cells transfected with siSCR or siRNAs against *NCL*, and relative quantification showing the percentage of mitotic cells identified for each type of alignment. Three independent replicates were performed, at least 50 cells per sample were analyzed. *$p<0.05$; **$p<0.01$; ***$p<0.001$. two-tailed unpaired t-test. (c) Tumors dissected from xenografts of *lncREST* KD and KO cells. (d) Apoptosis analysis of HCT116 cells transfected with CTRL or *lncREST* targeting LNA GapmeRs, measured by annexin V staining. Graph shows mean ± SD of two independent experiments. For b and d Source data are provided as a Source Data file.
