# Supplementary material for: The chromatin-associated lncREST ensures effective replication stress response by promoting the assembly of fork signaling factors
Source: Nat Commun. 2024 Feb 1;15:978. doi: 10.1038/s41467-024-45183-5 (PMC10834948; doi:10.1038/s41467-024-45183-5)
Supplement: Supplementary file 3 — Description of Additional Supplementary Files [file 41467_2024_45183_MOESM3_ESM.pdf]

Description of Additional Supplementary Files

File name: Supplementary data 1
Description: List of differentially expressed genes in total and chromatin fraction after treatment with hydroxyurea in HCT116 colon cancer cells

File name: Supplementary data 2
Description: List of proteins identified by mass spectrometry analysis in lncREST pulldown samples and control LacZ

File name: Supplementary data 3
Description: List of antibodies, primers, siRNAs, sgRNAs, LNA GapmeRs, and probes sequences used in this study.
