# Supplementary material for: The chromatin-associated lncREST ensures effective replication stress response by promoting the assembly of fork signaling factors
Source: Nat Commun. 2024 Feb 1;15:978. doi: 10.1038/s41467-024-45183-5 (PMC10834948; doi:10.1038/s41467-024-45183-5)
Supplement: Supplementary file 7 — Reporting Summary [file 41467_2024_45183_MOESM7_ESM.pdf]

Corresponding author(s): Maite Huarte, Luisa Statello

Last updated by author(s): Dec 20, 2023

Reporting Summary

Nature Portfolio wishes to improve the reproducibility of the work that we publish. This form provides structure for consistency and transparency in reporting. For further information on Nature Portfolio policies, see our Editorial Policies and the Editorial Policy Checklist.

Statistics

For all statistical analyses, confirm that the following items are present in the figure legend, table legend, main text, or Methods section.

n/a	Confirmed	
☐	☒	The exact sample size (*n*) for each experimental group/condition, given as a discrete number and unit of measurement
☐	☒	A statement on whether measurements were taken from distinct samples or whether the same sample was measured repeatedly
☐	☒	The statistical test(s) used AND whether they are one- or two-sided<br>*Only common tests should be described solely by name; describe more complex techniques in the Methods section.*
☒	☐	A description of all covariates tested
☒	☐	A description of any assumptions or corrections, such as tests of normality and adjustment for multiple comparisons
☐	☒	A full description of the statistical parameters including central tendency (e.g. means) or other basic estimates (e.g. regression coefficient) AND variation (e.g. standard deviation) or associated estimates of uncertainty (e.g. confidence intervals)
☐	☒	For null hypothesis testing, the test statistic (e.g. *F*, *t*, *r*) with confidence intervals, effect sizes, degrees of freedom and *P* value noted<br>*Give P values as exact values whenever suitable.*
☒	☐	For Bayesian analysis, information on the choice of priors and Markov chain Monte Carlo settings
☒	☐	For hierarchical and complex designs, identification of the appropriate level for tests and full reporting of outcomes
☒	☐	Estimates of effect sizes (e.g. Cohen's *d*, Pearson's *r*), indicating how they were calculated

Our web collection on statistics for biologists contains articles on many of the points above.

Software and code

Policy information about availability of computer code

Data collection	Image Studio Lite 5.2 was used to collect western blot Data; QuantStudio Real-Time PCR software v1.6.1 was used to collect quantitative PCR data; BD FACSDiva v 8.0.1 was used to collect flow citometer data; Cytexpert 2.3 was used to collect cell cycle data
Data analysis	GraphPAd Prism 8.0.2 software was used to generate graphs<br>R package ggplot2 was used to generate other plots (https://cran.r-project.org/web/packages/ggplot2/index.html)<br>Image J from Fiji processing package was used to analyze immunofluorescence and FISH images<br>Image Studio Lite 5.2 software was used to analyze western blot data<br>FlowJo v10 was used to analyze apoptosis data; Cytexpert 2.3 was used to analyze cell cycle data<br>RNA-seq sequences were trimmed with Trimmomatic (v.0.38), aligned with STAR (v.2.6.1), assigned to genes with featureCounts (v.1.6.3) and differentially expressed genes were assessed with DESeq2 and edgeR in R/Bioconductor.

For manuscripts utilizing custom algorithms or software that are central to the research but not yet described in published literature, software must be made available to editors and reviewers. We strongly encourage code deposition in a community repository (e.g. GitHub). See the Nature Portfolio guidelines for submitting code & software for further information.

Data

Policy information about availability of data

All manuscripts must include a data availability statement. This statement should provide the following information, where applicable:

- Accession codes, unique identifiers, or web links for publicly available datasets
- A description of any restrictions on data availability
- For clinical datasets or third party data, please ensure that the statement adheres to our policy

ChIP data used to identify peaks in lncREST promoter were retrieved form the following sources, and are found in ChIP atlas (http://chip-atlas.org): ATF3 (SRX1384025, GSM1917770), Zhao J, Li X, Guo M, Yu J et al. The common stress responsive transcription factor ATF3 binds genomic sites enriched with p300 and H3K27ac for transcriptional regulation. BMC Genomics 2016 May 4;17:335.
BRD4: (SRX1569429, GSM2058664),Baranello L, Wojtowicz D, Cui K, Devaiah BN et al. RNA Polymerase II Regulates Topoisomerase 1 Activity to Favor Efficient Transcription. Cell 2016 Apr 7;165(2):357-71. PMID: 27058666.
Myc: (SRX2734626, GSM2576763), Dejure FR, Royla N, Herold S, Kalb J et al. The MYC mRNA 3'-UTR couples RNA polymerase II function to glutamine and ribonucleotide levels. EMBO J 2017 Jul 3;36(13):1854-1868. PMID: 28408437.
P53: (SRX1384027, GSM1917772), Zhao J, Li X, Guo M, Yu J et al. The common stress responsive transcription factor ATF3 binds genomic sites enriched with p300 and H3K27ac for transcriptional regulation. BMC Genomics 2016 May 4;17:335.
E2F1: (SRX150556, GSM935477), ENCODE Project Consortium. An integrated encyclopedia of DNA elements in the human genome. Nature 2012 Sep 6;489(7414):57-74. PMID: 22955616
E2F6: (SRX190222, GSM1010766), Gertz J, Savic D, Varley KE, Partridge EC et al. Distinct properties of cell-type-specific and shared transcription factor binding sites. Mol Cell 2013 Oct 10;52(1):25-36. PMID: 24076218.
FOXM1: (SRX190187, GSM1010731), Gertz J, Savic D, Varley KE, Partridge EC et al. Distinct properties of cell-type-specific and shared transcription factor binding sites. Mol Cell 2013 Oct 10;52(1):25-36. PMID: 24076218
ChIP-seq public data from HCT116 cells treated with DNA damaging agent 5-FU were obtained from GEO series GSE58507.
Repliseq data on HCT116 cells were obtained from 'Zhao PA, Sasaki T, Gilbert DM. High-resolution Repli-Seq defines the temporal choreography of initiation, elongation and termination of replication in mammalian cells. Genome Biol 2020 Mar 24;21(1):76. PMID: 32209126', GEO series GSE137764.
Own-generated RNA-seq data have been deposited in Gene Expression Omnibus (GEO) under accession number GSE229870.

Research involving human participants, their data, or biological material

Policy information about studies with human participants or human data. See also policy information about sex, gender (identity/presentation), and sexual orientation and race, ethnicity and racism.

Reporting on sex and gender	N/A
Reporting on race, ethnicity, or other socially relevant groupings	N/A
Population characteristics	N/A
Recruitment	N/A
Ethics oversight	N/A

Note that full information on the approval of the study protocol must also be provided in the manuscript.

Field-specific reporting

Please select the one below that is the best fit for your research. If you are not sure, read the appropriate sections before making your selection.

☒ Life sciences ☐ Behavioural & social sciences ☐ Ecological, evolutionary & environmental sciences

For a reference copy of the document with all sections, see nature.com/documents/nr-reporting-summary-flat.pdf

Life sciences study design

All studies must disclose on these points even when the disclosure is negative.

Sample size	No statistical method was performed to establish sample size. The experiments were performedthree or four biological replicates to derive statistical significance, according to standards.
Data exclusions	No data were excluded from the analysis
Replication	All the experiments were reproduced in at least two independent replicates, but mostly three or more times. The results from biological replicates were all reproduced.

Randomization	All the experiments performed in vitro were done using cells seeded and treated in parallel for the different conditions in each replicate; for in vivo experiments, allocation of mice to experimental groups was random.
Blinding	Blinding was performed for the acquisition and analysis of DNA fiber assay immunofluorescence, RNA FISH and immunofluorescence of mitosis; For immunofluorescence and comet assay acquisition blinding was not performed as the same microscope setting were used for each type of experiment following a pilot experiment. However, blinding was performed for the analysis of immunofluorescnce experiments and comet assays.

Reporting for specific materials, systems and methods

We require information from authors about some types of materials, experimental systems and methods used in many studies. Here, indicate whether each material, system or method listed is relevant to your study. If you are not sure if a list item applies to your research, read the appropriate section before selecting a response.

Materials & experimental systems

n/a	Involved in the study
☐	☒ Antibodies
☐	☒ Eukaryotic cell lines
☒	☐ Palaeontology and archaeology
☐	☒ Animals and other organisms
☒	☐ Clinical data
☒	☐ Dual use research of concern
☒	☐ Plants

Methods

n/a	Involved in the study
☒	☐ ChIP-seq
☐	☒ Flow cytometry
☒	☐ MRI-based neuroimaging

Antibodies

Antibodies used

Anti-phospho histone H2A.X Millipore, # 05-636
ATR (phospho Thr1989) Gene Tex, #GTX128145
RPA70/RPA1 Cell Signaling, #2267
RPA 32 kDa subunit (9H8) Santa Cruz, sc-56770
Histone H3 Abcam, #ab10799
ssDNA Millipore, #MAB3034
BrdU antibody BU 1/75 (ICR1) Abcam, #ab6326
BrDU (B44) BD, #347580
PCNA Santa Cruz, sc-56
RAD51 Santa Cruz, sc-53428
GAPDH (14C10) Cell Signaling, #3683
NCL (C23) Santa Cruz, sc-17826
alpha-tubulin Millipore, #T6199
P-CENP-A (S7) Cell Signaling, #2187
H2AX Santa Cruz #sc-517336
TR, Santa Cruz #sc-515173
CHK1 (G4), Santa Cruz sc-8404
pCHK1(S345) Cell signaling #2341
Normal mouse IgG Santa Cruz, sc-2025
Alexa fluor 488 goat anti-mouse Invitrogen, #A21121
Alexa fluor 647 goat anti-mouse Invitrogen, #A21241
Alexa fluor 594 goat anti-Rat Invitrogen, #A11007
Anti-Fluorescein-POD, Fab fragments Roche, #11426346910

Validation

All the available information on antibody validation can be found by searching the manufacturer's websites.
Anti-phospho histone H2A.X Millipore, # 05-636: https://www.merckmillipore.com/ES/es/product/Anti-phospho-Histone-H2A.X-Ser139-Antibody-clone-JBW301,MM_NF-05-636. Validated in ChIP, ICC, IF, WB.
ATR (phospho Thr1989) Gene Tex, #GTX128145: https://www.genetex.com/Product/Detail/ATR-phospho-Thr1989-antibody/GTX128145. Validated inWB, ICC/IF, IHC-P, IP.
RPA70/RPA1 Cell Signaling: #2267: https://www.cellsignal.com/datasheet.jsp?productId=2267&images=1&size=A4. Validated in WB, IP, IF-IC, FC-FP.
RPA 32 kDa subunit (9H8) Santa Cruz, sc-56770: https://datasheets.scbt.com/sc-56770.pdf
H3 ab1791 (Abcam): https://www.abcam.com/histone-h3-antibody-nuclear-marker-and-chip-grade-ab1791.html
ssDNA Millipore, #MAB3034: https://www.merckmillipore.com/ES/es/product/Anti-DNA-Antibody-single-stranded-clone-16-19,MM_NF-MAB3034. Published in Terret et al. 2009. Nature
BrdU antibody BU 1/75 (ICR1) Abcam, #ab6326: https://www.abcam.com/products/primary-antibodies/brdu-antibody-bu175-icr1-proliferation-marker-ab6326.html. Published in Terret et al. 2009. Nature.
BrDU (B44) BD, #347580: https://www.bdbiosciences.com/content/bdb/paths/generate-tds-document.es.347580.pdf. Published in Terret et al. 2009. Nature.
PCNA Santa Cruz, sc-56: https://www.scbt.com/p/pcna-antibody-pc10.
RAD51 Santa Cruz, sc-53428: https://datasheets.scbt.com/sc-53428.pdf.
GAPDH (HRP Conjugate) 3683 (Cell Signalling): https://www.cellsignal.com/products/antibody-conjugates/gapdh-14c10-rabbit-mab-

hrp-conjugate/3683.
NCL (C23) Santa Cruz, sc-17826: https://datasheets.scbt.com/sc-17826.pdf.
alpha-tubulin Millipore, #T6199: https://www.sigmaaldrich.com/specification-sheets/260/142/T6199-BULK________SIGMA____.pdf
P-CENP-A (S7) Cell Signaling, #2187: https://www.cellsignal.com/datasheet.jsp?productId=2187&images=1&size=A4
H2AX Santa Cruz #sc-517336: https://www.scbt.com/es/p/histone-h2a-x-antibody-938ct5-1-1;
ATR, Santa Cruz #sc-515173: https://www.scbt.com/p/atr-antibody-c-1?requestFrom=search;
CHK1 (G4), Santa Cruz sc-8404: https://www.scbt.com/p/chk1-antibody-g-4?requestFrom=search;
pCHK1(S345) Cell signaling #2341: https://www.cellsignal.com/products/primary-antibodies/phospho-chk1-ser345-antibody/2341

Eukaryotic cell lines

Policy information about cell lines and Sex and Gender in Research

Cell line source(s): HCT116 (CCL-247), H358 (CRL-5807), HEK-293T (CRL-3216), LoVo (CCL-229), RKO (CRL-2577), A549 (CRM-CCL-185), RPE1 and U2OS (HTB-96) were purchased from the American Type Culture Collection (ATCC). COV362, purchased from MERK, were from European Collection of Authenticated Cell Culture (ECACC). JHH6 were obtained from Puri Fortes lab at CIMA (Centre for Applied Medical Research), TIG-3 cells were from Anders Lund's lab at BRIC, University of Copenhagen.

Authentication: None of the cell lines were authenticated

Mycoplasma contamination: All cell lines were tested and resulted negative for mycoplasma contamiantion

Commonly misidentified lines (See ICLAC register): no commonly misidentified cell lines were used in the study

Animals and other research organisms

Policy information about studies involving animals; ARRIVE guidelines recommended for reporting animal research, and Sex and Gender in Research

Laboratory animals: 6-7 weeks-old female and male BALB/cA-Rag2−/−γc−/− immunodeficient mice

Wild animals: The study did not involve wild animals

Reporting on sex: Male and female mice were used in equal proportion for this study

Field-collected samples: The study does not involve field-collected samples

Ethics oversight: The ethical protocol was submitted to the spanish organization 'Instituto de salud publica y laboral de Navarra'. The protocol was approved by the Comité de Ética para la experimentación animal of the University of Navarra with the following code: 006-20.

Note that full information on the approval of the study protocol must also be provided in the manuscript.

Flow Cytometry

Plots

Confirm that:

- ☐ The axis labels state the marker and fluorochrome used (e.g. CD4-FITC).
- ☐ The axis scales are clearly visible. Include numbers along axes only for bottom left plot of group (a 'group' is an analysis of identical markers).
- ☐ All plots are contour plots with outliers or pseudocolor plots.
- ☒ A numerical value for number of cells or percentage (with statistics) is provided.

Methodology

Sample preparation: For cell cycle analysis HCT116 cells were grown, treated and detached with trypsin. 1x106 cells were centrifuged at 1200 rpm for 5 min and resuspended in 200 µl Ethanol 70% overnight at 4ºC. Fixed cells were washed with PBS and resuspended in 250 µl PBS adding 5µl of 10mg/ml RNAse A solution for 1h at 37ºC. After adding propidium iodide cells were analyzed. For apoptosis assessment, HCT116 cells were grown, treated detached with trypsin. Apoptosis was assessed by Annexin V and 7-AAD staining using the Apoptosis Detection Kit I 791 (BD biosciences).

Instrument: For cell cycle analysis: CytoFLEX XL (Beckman Coulter). For apoptosis analysis: FACS Canto II (BD Biosciences)

Software: Acquisition software: FACSDiva (BD Biosciences), analysis with Cytexpert 2.3 for cell cycle, FlowJo software for apoptosis.

Cell population abundance: Following single cells gating, about 25% of the total events was used for evaluating the final cell population abundance. For cell cycle analysis, about 80-90% of these cells was further gated as positive to propidium iodide to define cell cycle

populations G1, S and G2; for apoptosis analysis, Annexin V and 7-AAD positive cells (early and late apoptosis) were 20-40% of the single cells, based on the type of treatment.

Gating strategy

All samples were initially gated using forward scatter and side scatter to identify events corresponding to cells, then single cells were gated using forward scatter height versus area. For cell cycle analysis, positive cells to propidium iodide were defined using B690-PC5.5A area vs. width. For apoptosis analysis, single cells where gated using PE (for Annexin V) vs PerCP-Cy5-5 (for 7-AAD). Gating were set using different control samples as untreated cells, and cells incubated only with Annexin V or 7-AAD antibodies.

☐ Tick this box to confirm that a figure exemplifying the gating strategy is provided in the Supplementary Information.
